# Clustering Heatmap for Visualizing and Exploring Complex and High-dimensional Data Related to Chronic Kidney Disease

**DOI:** 10.3390/jcm9020403

**Published:** 2020-02-02

**Authors:** Cheng-Sheng Yu, Chang-Hsien Lin, Yu-Jiun Lin, Shiyng-Yu Lin, Sen-Te Wang, Jenny L Wu, Ming-Hui Tsai, Shy-Shin Chang

**Affiliations:** 1 Department of Family Medicine, Taipei Medical University Hospital, Taipei 110, Taiwan; molytrigger@gmail.com (C.-S.Y.);; 2 Department of Family Medicine, School of Medicine, College of Medicine, Taipei Medical University, Taipei 110, Taiwan; 3 Preventive health center, Taipei Medical University Hospital, Taipei 110, Taiwan

**Keywords:** heatmap, clustering, multivariate statistical analysis, chronic kidney disease, risk factors

## Abstract

Background: Preventive medicine and primary health care are essential for patients with chronic kidney disease (CKD) because the symptoms of CKD may not appear until the renal function is severely compromised. Early identification of the risk factors of CKD is critical for preventing kidney damage and adverse outcomes. Early recognition of rapid progression to advanced CKD in certain high-risk populations is vital. Methods: This is a retrospective cohort study, the population screened and the site where the study has been performed. Multivariate statistical analysis was used to assess the prediction of CKD as many potential risk factors are involved. The clustering heatmap and random forest provides an interactive visualization for the classification of patients with different CKD stages. Results: uric acid, blood urea nitrogen, waist circumference, serum glutamic oxaloacetic transaminase, and hemoglobin A1c (HbA1c) were significantly associated with CKD. CKD was highly associated with obesity, hyperglycemia, and liver function. Hypertension and HbA1c were in the same cluster with a similar pattern, whereas high-density lipoprotein cholesterol had an opposite pattern, which was also verified using heatmap. Early staged CKD patients who are grouped into the same cluster as advanced staged CKD patients could be at high risk for rapid decline of kidney function and should be closely monitored. Conclusions: The clustering heatmap provided a new predictive model of health care management for patients at high risk of rapid CKD progression. This model could help physicians make an accurate diagnosis of this progressive and complex disease.

## 1. Introduction

Chronic kidney disease (CKD) poses a substantial challenge to global health policy because of its health and economic burden [1]. Primary health care is vital for CKD because of the high global prevalence (11%–13%) of underdiagnosed and undertreated patients with stage 3 CKD [2]. In Taiwan, the prevalence of CKD is as high as 11.93% (approximately 2.74 million patients), resulting in a potentially high mortality rate because of cardiovascular diseases and accounting for tremendous health care expenditures [3]. Therefore, early identification of the risk factors for CKD is critical for preventing kidney damage and adverse outcomes.

Identification and staging of CKD rely on the measurement of glomerular filtration rate (GFR) and albuminuria. The calculation of actual GFR by measurement of external filtration markers is cumbersome and impractical. Moreover, clinical studies have found that external GFR markers are not reliable predictors of risk of advanced CKD because of the inconsistency in their levels at different stages of CKD [4,5,6,7,8].

Recently, machine learning techniques have showed success in prediction and diagnosis of numerous critical diseases. In fact, one study found that CKD can be diagnosed with an accuracy of 0.98, using supervised machine learning technique [9]. However, to the best of our knowledge, there is still no attempt to classify and visualize CKD staging via machine learning technique. Given that GFR measurement can be cumbersome and inconsistent, we aimed to use unsupervised learning technique, hierarchical clustering with heatmap visualization to classify the CKD staging of patients. The results of our study may identify patients who are categorized as having early stage CKD but have physiological parameters similar to those with advanced-stage CKD. Thus, clinicians could provide active health intervention to this group to minimize their risk of advanced renal disease.

## 2. Experimental Section

### 2.1. Study Design

This single-center, retrospective cohort study assessed the ability of the clustering heatmap to classify and predict the risk of CKD in participants who underwent a self-paid health examination at the Health Management Center (HMC) of Taipei Medical University Hospital (TMUH).

### 2.2. Setting

The study was conducted at TMUH, and patients’ electronic medical records were reviewed. TMUH is a private, 800-bed teaching hospital in Taiwan. HMC of the TMUH receives 50–60 visits each day. The Institutional Review Board of TMUH approved this study (TMU-JIRB No.: N201903080), which was conducted in accordance with the original and amended Declaration of Helsinki. IRB approval was obtained for this project before starting data collection and informed consent was waived by the IRB owing to the retrospective nature of the study.

### 2.3. Inclusion and Exclusion Criteria

The population of interest was participants who underwent a self-paid health examination at the HMC of TMUH. To be included in the analysis, the self-paid health examination participants must have undergone a self-paid health examination comprising of an abdominal transient elastography inspection using FibroScan 502 Touch (Echosens, Paris, France) and urine test from March 2015 to December 2019. Exclusion criteria included, participants the age of participants ≤ 18 years old, and participants that do not have follow-up urine test, within 3 months when the first urine test is found to be abnormal.

### 2.4. Data

All participants undertook the regular processes of HMC. The participants were interviewed by well-trained personnel, who verified the correctness of the participants’ self-completed questionnaire on demographics, existing medical conditions, and use of medication. In addition, the personnel confirmed adherence to health examination prerequisites (e.g., overnight fasting for at least 8 h) for the package chosen by the participants. Those found to have not followed the prerequisites were suggested to book another appointment. Then, anthropometrics (weight, height, waist circumference, and arterial pressures) were measured. The instruments were regularly calibrated per the manufacturer’s specifications. The samples of blood, urine, and specimens required per the chosen package were collected for laboratory tests. Regular laboratory test items included alpha-fetoprotein, hemoglobin A1c (HbA1c), serum glutamic oxaloacetic transaminase (GOT), serum glutamic-pyruvic transaminase (GPT), uric acid (UA), creatinine, blood urea nitrogen (BUN), total protein, albumin, globulin, albumin/globulin ratio (A/G), total bilirubin, direct bilirubin, alkaline phosphatase, gamma-glutamyl transpeptidase (r-GT), total cholesterol, low-density lipoprotein cholesterol (LDL), high-density lipoprotein cholesterol (HDL), LDL/HDL ratio, total cholesterol/HDL ratio, triglycerides, fasting blood sugar, and thyroid-stimulating hormone (TSH). Urine specimens were obtained in the morning and scheduled to avoid menstrual periods and check proteinuria, hematuria, red blood cell cast, white blood cell cast, and other urine sediment abnormalities. A repeated check on urine specimens was performed within 3 months, if an abnormal urine test is obtained. For each FibroScan (502 Touch; Echosens, Paris, France) inspection, two scores were reported: controlled attenuation parameter (CAP) score and liver stiffness parameter (E score) [10,11,12,13].

### 2.5. Definitions of Measurement Cutoffs and Calculations

Body mass index (BMI) categories were defined according to the ranges established for Asian populations by the Ministry of Health and Welfare of Taiwan as follows: obesity, ≥27 kg/m^2^; overweight, 24–26.9 kg/m^2^; and normal weight, <23.9 kg/m^2^ [14]. The cutoff for waist circumference for abdominal obesity was ≥90 cm for men and ≥80 cm for women, using the Asian-specific cutoff points established by the International Diabetes Federation [15]. Estimated GFR (eGFR) was calculated using equations for the Modification of Diet in Renal Disease (MDRD) formula for Chinese patients as follows: 175 × (Scr) ^−1.234^× (age) ^−0.179^ × 0.79 (if female) [16]. In this study, CKD was classified into five stages using Kidney Outcomes Quality Initiative (KDOQI) [17] guidelines using thresholds of eGFR within the CKD range and/or evidence of structural renal changes, e.g., proteinuria. In our study, we used equations for the Modification of Diet in Renal Disease for Chinese patients with CKD [16] measured in the following manner: 175 × (Scr) ^−1.234^ × (Age) ^−0.179^ × 0.79 (if female). The definition of the five stages of CKD was stage-1 (eGFR > 90 mL/min/1.73 m^2^ with serial proteinuria positive); stage-2 (eGFR 60–89 mL/min/1.73 m^2^ with serial proteinuria positive); stage-3 (eGFR 30–59 mL/min/1.73 m^2^; Stage-4 (eGFR 29–15 mL/min/1.73 m^2^); and stage-5 (eGFR < 15 mL/min/1.73 m^2^). Serial proteinuria was defined as two positive proteinuria in two separate urine tests over a period of 3 months.

### 2.6. Statistical Analysis

Statistical analysis was conducted using R (version 3.6.1) or SPSS (version 17.0) software. In Table 1, the baseline characteristics of the enrollees were described, and the *p*-value denotes comparison between CKD stage 1 and CKD stage 2–5 participants. Binary variables were presented as a frequency and percentage and were compared using the Chi-squared test or Fisher’s exact test. The Mann–Whitney U-test, a nonparametric test, was used to compare the medians of continuous variables. In Table 2, multivariate logistic regression was conducted to assess the significance of clinical data, and *p*-value denotes whether the variable is statistically significant. In addition, variance inflation factor (VIF) was used to check multicollinearity [18,19]. In all analysis, *p*-value < 0.05 was considered statistically significant.

#### 2.6.1. Receiver Operating Characteristic Curve

Receiver operating characteristic (ROC) curves were used to illustrate the diagnostic ability of machine learning classification. Area under the curve (AUC), true-positive rate (also called sensitivity or recall), and false-positive rate (related to specificity) are shown in a graphical plot [20].

#### 2.6.2. Random Forest

Random decision forest, an ensemble learning method for classification, regression, or other applications based on a decision tree structure, was used to extract the most relevant variables from those biomarkers. The random forest model created multiple decision trees and then combined the output generated by each decision tree [21,22]. This approach removed the bias that a decision tree model might introduce in the system, thus considerably improving the predictive power. All procedures in the random forest model were conducted in the R package “randomForest” [23].

#### 2.6.3. Missing Values

Data with missing values were regulated by an expectation–maximization algorithm, which was an iterative procedure and preserved the relationship with other variables. After deletion of variables with missing values >10% of the sample size, 23 variables were retained [24].

#### 2.6.4. Multivariate Analysis

Multivariate analysis, which involved observation and analysis of more than one statistical outcome variable simultaneously, was employed as the analysis of large multivariable data sets was a major challenge for life science research. Multivariate analysis has been made much easier with inexpensive, fast computers and powerful analytical software. The application of multivariate analysis included dimensionality reduction, clustering, and variable selection. Here, a heatmap and hierarchical clustering were used [25].

#### 2.6.5. Heatmap and Clustering

A heatmap was used to visualize the pattern of the medical variables generated through classification. The heatmap, a graphical representation of data with the individual values contained in a matrix, was represented as grids of colors plus clustering on both rows and columns. Clustering was applied to group a set of patients according to their health examination data. Participants were divided into different clusters using Ward’s hierarchical clustering method, and the patterns of their biomarkers were shown in colors in the center of the heatmap. The heatmap was available from the package “gplot” as an enhanced version or its basic function stats in R [26,27,28,29,30,31].

## 3. Results

**Figure 1:**
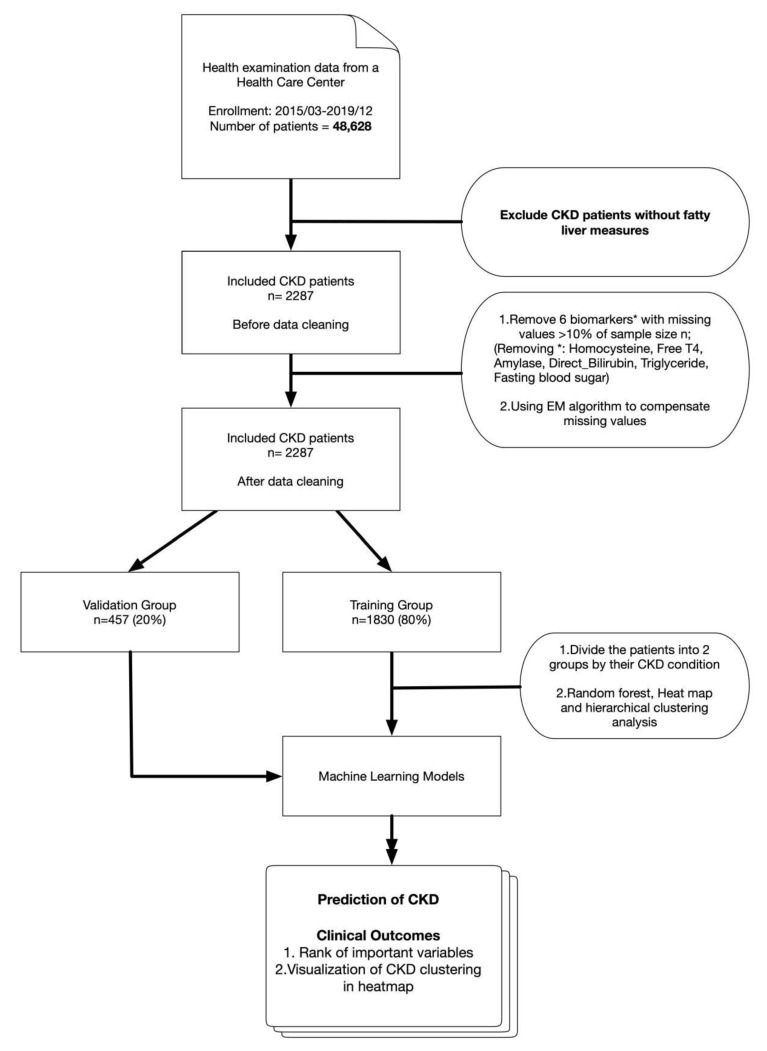
A flowchart of the step-by-step procedure from data collection and preprocessing to statistical analyses.

A total of 2287 participants were enrolled. The whole procedure from data collection to statistical analysis and outcome is shown in Figure 1. After a series of data-cleaning procedures, the participants were stratified into three groups (stage 1, stage 2, and stage 3–5) and compared on Table 1. A higher percentage of female was observed in stage 1 (59.3%) as compared to stage 2 (25.9%) and stage 3–5 (31.2%). As expected, stage 1 CKD participants were generally younger and healthier than stage 2 and stage 3–5 participants.

Variables that had prominent effects on CKD were assessed by multivariate logistic regression (Table 2). Two biomarkers (BUN, odds ratios of 1.229 and UA, odds ratios of 1.478) related to kidney function and one biomarker related to metabolic syndrome (HbA1C, odds ratios of 1.248) were found to have substantial positive association with CKD status. Other metabolic syndrome markers that exhibited significant positive association with CKD status were BMI, and WC. In addition, significant association was observed for three hepatic markers (GOT, GPT and CAPscore). VIF detected multicollinearity between cholesterol and LDL; hence, cholesterol was excluded from subsequent multivariate analyses.

**Figure 2:**
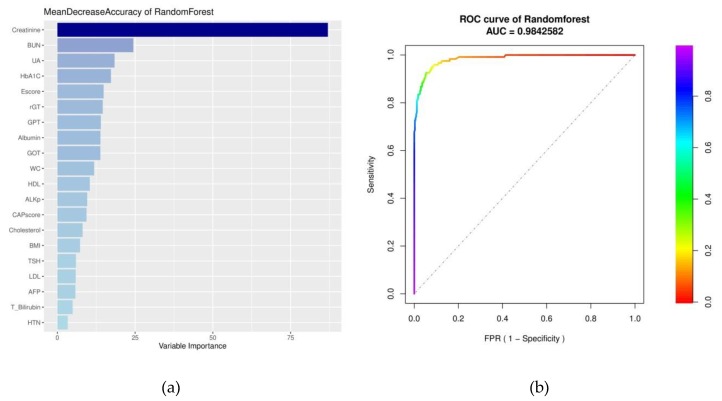
(**a**) Variable importance ordered by the accuracy of mean decrease in random forest. The leading variables obtained by random forest list in **a** with a darker blue; conversely, less prominent variables are indicated in a lighter blue. (**b**) area under the ROC curve of random forest. The rainbow bar indicates the value of specificity in the false-positive rate.

For every variable, the weights of importance measured using random forest are depicted in Figure 2a. The top rank of variables, which had leading scores of mean decrease accuracy in random forest, were similar to the multivariable analysis, Creatinine (87.2%), BUN (24.4%), UA (18.3%), HbA1c (17.2%), Escore (15%), γGT (14.7%), and GPT (14.2%). The AUC of the random forest model using ROC was approximately 0.984 (Figure 2b).

**Figure 3:**
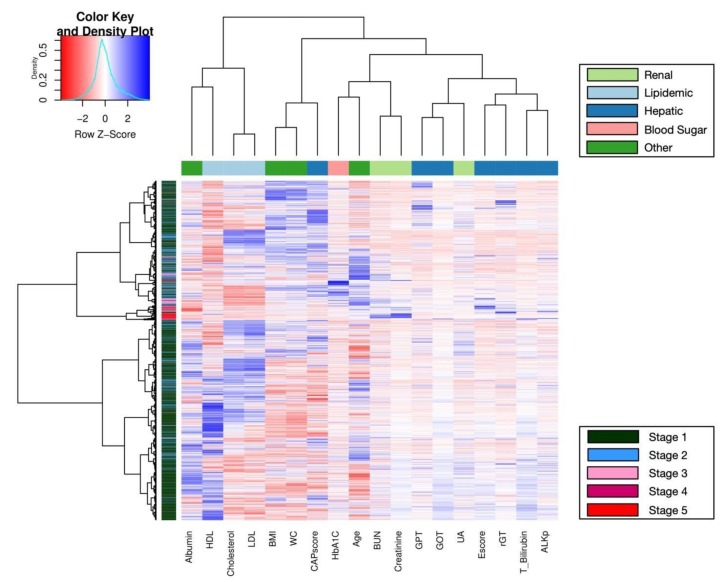
A clustering heatmap illustrating the classification of chronic kidney disease (CKD) in health examination data. Both the rows of CKD patients and the columns of biomarkers have been clustered, respectively; row data is also normalized into Z-score, simultaneously. Moreover, the mapping grids in the center have been colored according to their z scores.

Finally, multivariate analysis using clustering heatmaps described the classification of CKD stages and related biomarkers (Figure 3). In the row clustering step, most patients with early CKD (stage 1 in dark green; stage 2 in sky blue) were grouped in the lower cluster, whereas most patients with more advanced CKD (stage 3 in pink; stage 4 in red violet; stage 5 in red) were grouped into the center clusters. In addition, every biomarker was clustered into different subgroups on the column side according to their color patterns in the center grids of heatmap. Hepatic biomarkers (blue) were grouped together to the right side with renal biomarkers (light green) because their patterns were more similar than the others. Metabolic (green and salmon) and lipid biomarkers (light blue) were grouped into the left cluster as their patterns were more similar. HDL had an opposite color pattern because higher HDL indicates a healthy state. Heatmaps provided a systematic and clustered visualization of the analyzed data, facilitating monitoring of CKD patients according to their health examination data (Figure 3).

## 4. Discussion

Traditionally, CKD stages have been defined by only eGFR, an approach that is neither punctilious nor precise, because CKD is a progressive and complex disease. This is especially complicated in early stage CKD patients with a steep decline in their renal function. Therefore, it is challenging for clinicians to predict the progression of CKD and to identify early patient groups at risk of rapid deterioration within the same stage of CKD. Using random forest analysis, we found that the significant variables that played principal roles in CKD were Creatinine, BUN and UA as nephritic factors; HbA1C as blood glucose factors; Escore, γGT and GPT as hepatic factors. In addition, this study found that a clustering heatmap was a practical and accurate method to classify CKD patients into several clusters as well as perform multigroup analysis. In addition, heatmaps that involved plenty of biomarkers were more dynamic, equitable, and comprehensive than a fixed criterion measured by MDRD considering only age, race, sex, and creatinine.

There is a paucity of studies that used machine learning to predict CKD, and we found one study that have very similar study design and results like ours. Using hospital data from India, Anusorn Charleonnan et al. found that the accuracy of prediction in CKD reached 0.98, very similar to what we have found (Figure 2b) [9]. However, Charleonnan et al. had not discussed and rank the significant factors, which contributed to prediction of CKD. Our study not only showed the importance of risk factors in predicting CKD by ensemble learning model, but also discussed the outcomes and meanings in clinical medicine. Furthermore, we have applied unsupervised learning technique, hierarchical clustering with heatmap visualization to classify and monitor patients in different groups. This approach might be useful for prediction and prevention of other chronic diseases that involved staging.

For heatmaps, we used Euclidean distance as the distance measure and Ward’s method for unsupervised clustering. The clustering may be upgraded using other measures or algorithms. In addition, the heatmap may be more robust and dynamic with adequate sample size of patients at different stages or by using other specific and remarkable variables. Accordingly, new patients could be assessed using this clustering heatmap system based on their health conditions; if the new patients, especially those in early stages of CKD, had patterns similar to those in advanced CKD groups, precautionary treatment could be administered. In the clustering of medical factors, GPT, GOT and UA were in the same cluster with a similar pattern, whereas HDL, being a sign of good health, had the opposite pattern.

The CAP and E score by FibroScan are accurate non-invasive methods for assessing liver steatosis and fibrosis in patients with NAFLD [32]. CKD and NAFLD share a common pathological pathway and many important cardiometabolic risk factors. Moreover, the presence of pathophysiological interrelationships between the liver and kidney is well established and is supported by the presence of the hepatorenal syndrome, which may occur in patients with decompensated cirrhosis, regardless of its etiology. Patients with NAFLD had a significantly higher risk of incident CKD than those without NAFLD. Patients with more ‘severe’ NAFLD were also more likely to develop incident CKD [33]. NAFLD per se affects CKD through lipoprotein metabolism and hepatokine secretion, and conversely, targeting the renal tubule by sodium-glucose cotransporter 2 inhibitors can improve both CKD and NAFLD [34].

The result on hyperuricemia can be explained by association with the acceleration of GFR decline and CKD progression [35,36,37]. Previous research found that dyslipidemia in CKD is characterized by elevated triglycerides and decreased and dysfunctional HDL [38], and our results suggest that hypertension, hyperglycemia, and their combination may be associated with the incidence of CKD [39,40,41]. The association with HbA1C can be explained by diabetic patients being strongly associated with both albuminuria and reduced eGFR, and in the US it was found that diabetic patients had a substantially higher prevalence of CKD [42].

There are several limitations in this study. First, this study investigated only health-conscious participants that underwent a self-paid health examination, and the number of patients with advanced CKD may be underrepresented. Having approximately the same percentage of patients across different CKD stages will establish a much better model, and hence repeating this study by selectively including more advanced CKD patients is one of the future goals of this study. Second, this is a retrospective study, and confounding cannot be excluded. Hence, the subsequent step is to conduct a large prospective study to test the accuracy and usefulness of the predictive model. Third, this study concerned mainly Han Chinese population residing in Taiwan, and the study results needs to be repeated and validated in other populations. Fourth, this study failed to include some new CKD diagnostics that may further improve the prediction of CKD staging. For example, it was recently found that salivary ferric ion reducing antioxidant parameter can distinguish patients with mildly to moderately decreased kidney function from those with severe renal impairment with an accuracy of 100% (AUROC) [43].

## 5. Conclusions

Using machine learning technologies, we have generated a new predictive model of health care management for patients within the high-risk CKD group. However, considering the retrospective nature of this study, a sufficiently powered prospective cohort study is needed to conclusively address the usefulness of this predictive model to help physicians make an accurate diagnosis of the staging of CKD, which changes over time.

## Figures and Tables

**Table 1:** Descriptive statistics and testing of medical variables in health examination data with chronic kidney disease.

	CKD Stage 1	CKD Stage 2	CKD Stage 3–5	
	*n*_1_ = 1715	*n*_2_ = 370	*n*_3_ = 202	
Factors	No. (%)	No. (%)	No. (%)	*p*-Value
Sex				
Female	1017 (59.3%)	96(25.9%)	63(31.2%)	<0.001
Male	698 (40.7%)	274(74.1%)	139(68.8%)	
Hypertension				
Normal	1299 (75.7%)	214(57.8%)	112(55.4%)	<0.001
High	416 (24.3%)	156(42.2%)	90(44.6%)	
Median (IQR)				
Age, years	42 (35–51)	53 (46–60.75)	63.5 (55.25–73)	<0.001
Albumin, g/dL	4.6 (4.4–4.8)	4.6 (4.5–4.8)	4.3 (3.8–4.5)	<0.001
BMI, kg/m^2^	23.4 (21.1–25.99)	24.7 (22.7–27.02)	25.05 (22.15–27.98)	<0.001
WC, cm	80.5 (73.5–88)	86 (80–92)	87.58 (82–95.75)	<0.001
AFP, ng/mL	2.35 (1.62–3.27)	2.62 (1.98–3.62)	2.69 (2.15–4.48)	<0.001
ALKp, IU/L	60 (50–72)	67 (55–79)	79.69 (65.68–96)	<0.001
GOT, IU/L	20 (17–24)	23 (20–28)	23.1 (19–34)	<0.001
GPT, IU/L	18 (13–28)	23 (17–33)	22 (15–35)	<0.001
T_Bilirubin, mg/dL	0.6 (0.4–0.8)	0.6 (0.5–0.8)	0.6 (0.4–0.9)	0.002
γGT, U/L	17 (12–27)	23 (17–34)	34.63 (21–70.90)	<0.001
CAPscore, dB/m	241 (208–281)	260 (227–301.8)	246 (203–296.5)	<0.001
Escore, kPa	4.3 (3.5–5.1)	4.6 (3.7–5.6)	7.9 (4.925–13.525)	<0.001
BUN, mg/dL	12 (10–14.34)	15 (13–18)	23.5 (16.78–38.82)	<0.001
Creatinine, mg/dL	0.7 (0.6–0.8)	1 (1–1.1)	1.7 (1.425–3.9)	<0.001
UA, mg/dL	5.1 (4.3–6.3)	6.3 (5.5–7.3)	6.6 (5.7–7.975)	<0.001
Cholesterol, mg/dL	187 (165–209)	191.5 (165–217)	169 (130.2–195)	<0.001
HbA1C, %	5.4 (5.2–5.6)	5.525 (5.3–5.9)	5.7 (5.3–6.3)	<0.001
HDL, mg/dL	54 (45–65)	49 (41–58)	46 (37–57.9)	<0.001
LDL, mg/dL	121 (102–143)	131 (102.2–152)	107 (80.25–128.75)	<0.001
TSH, μIU/mL	1.81(1.2–2.545)	2.075 (1.465–2.835)	2.009 (1.54–2.565)	<0.001

CKD, chronic kidney disease; BMI, body mass index; WC, waist circumference; AFP, alpha-fetoprotein; ALKp, alkaline phosphatase; GOT, serum glutamic-oxalocetic transaminase; GPT, serum glutamic-pyruvic transaminase; γGT, γ-Glutamyl transpeptidase; BUN, blood urea nitrogen; UA, uric acid; HDL, high-density lipoprotein; LDL, low-density lipoprotein; HbA1C, glycated hemoglobin; TSH, thyroid-stimulating hormone; IU, international units; U, μmol/min; μIU, micro-international units. The upper part includes categorical variables and the others are continuous variables, which are general physiological indices in the first section, hepatic indices and nephritic elements in the second section, and blood lipid and thyroid indices in the final section.

**Table 2:** Multivariate logistic regression analysis of whole biomarkers related to chronic kidney disease.

Factors	Odds Ratio	95% CI OR	VIF	ΔVIF	*p*-Value
BMI, kg/m^2^	0.904	(0.854, 0.958)	3.977	3.975	**0.001** ^*^
WC, cm	1.046	(1.023, 1.069)	4.170	4.169	**<0.001** ^*^
Cholesterol, mg/dL	0.983	(0.973, 0.994)	**10.598**	**Δ**	**0.002** ^*^
Hypertension	0.213	(0.654, 1.099)	1.123	1.122	0.213
HbA1C, %	**1.248**	(1.087, 1.434)	1.223	1.178	**0.002** ^*^
HDL, mg/dL	1.005	(0.994, 1.016)	1.945	1.334	0.363
LDL, mg/dL	1.016	(1.005, 1.028)	**10.286**	**1.077**	**0.004** ^*^
Albumin, g/dL	0.793	(0.536, 1.174)	1.355	1.364	0.247
ALKp, IU/L	1.001	(0.995, 1.006)	1.202	1.209	0.761
GOT, IU/L	1.030	(1.012, 1.050)	3.832	3.801	**0.001** ^*^
GPT, IU/L	0.977	(0.966, 0.988)	3.847	3.803	**<0.001** ^*^
γGT, U/L	1.002	(0.997, 1.006)	1.452	1.448	0.425
T_Bilirubin, mg/dL	1.088	(0.930, 1.273)	1.182	1.180	0.291
CAPscore, dB/m	1.003	(1.000, 1.006)	1.646	1.647	**0.044** ^†^
Escore, kPa	1.012	(0.985, 1.040)	1.622	1.595	0.393
AFP, ng/mL	1.000	(1.000, 1.000)	1.141	1.129	0.528
BUN, mg/dL	**1.229**	(1.190, 1.270)	1.063	1.062	**<0.001** ^*^
UA, mg/dL	**1.478**	(1.349, 1.619)	1.346	1.333	**<0.001** ^*^
TSH, μIU/mL	1.047	(1.004, 1.091)	1.016	1.015	0.031

* indicates the *p* value < 0.001, † indicates the *p*-value < 0.05. BMI, body mass index; WC, waist circumference; HbA1C, glycated hemoglobin; HDL, high-density lipoprotein; LDL, low-density lipoprotein; ALKp, alkaline phosphatase; GOT, serum glutamic-oxalocetic transaminase; GPT, serum glutamic-pyruvic transaminase; γGT, γ-Glutamyl transpeptidase; AFP, alpha-fetoprotein; BUN, blood urea nitrogen; UA, uric acid; TSH, thyroid-stimulating hormone; IU, international units; U, μmol/min; μIU, micro-international units. Chronic kidney disease is the dependent variable, and all the biomarkers are the independent variables in the logistic analysis. The odds ratio represents the exp(β), which is the exponential of the estimator in logistic regression with 95% confidence interval. In addition, the variance inflation factor (VIF) for each variable is calculated to check multicollinearity. Factors with high odds ratio or significant *p*-value are marked in bold. Factors with high VIF values are shaded. ΔVIF records the variance inflation factor (VIF) after removing the predictor variables with high VIF value.
